# NaCl-, protease-tolerant and cold-active endoglucanase from *Paenibacillus* sp. YD236 isolated from the feces of *Bos frontalis*

**DOI:** 10.1186/s40064-016-2360-9

**Published:** 2016-06-16

**Authors:** Mingjie Dong, Yunjuan Yang, Xianghua Tang, Jidong Shen, Bo Xu, Junjun Li, Qian Wu, Junpei Zhou, Junmei Ding, Nanyu Han, Yuelin Mu, Zunxi Huang

**Affiliations:** School of Life Science, Yunnan Normal University, Kunming, 650500 People’s Republic of China; Engineering Research Center of Sustainable Development and Utilization of Biomass Energy, Ministry of Education, Yunnan Normal University, No.1 Yuhua District, Chenggong, Kunming, 650500 Yunnan People’s Republic of China; Key Laboratory of Yunnan for Biomass Energy and Biotechnology of Environment, Kunming, 650500 Yunnan People’s Republic of China; Key Laboratory of Enzyme Engineering, Yunnan Normal University, Kunming, 650500 People’s Republic of China

**Keywords:** *Bos frontalis*, *Paenibacillus*, Endoglucanase, Heterologous expression, Enzyme characterization

## Abstract

*Bos frontalis*, which consumes bamboo and weeds, may have evolved unique gastrointestinal microorganisms that digest cellulase. A *Paenibacillus* sp. YD236 strain was isolated from *B. frontalis* feces, from which a GH8 endoglucanase gene, *pglue8* (1107 bp, 54.5 % GC content), encoding a 368-residue polypeptide (PgluE8, 40.4 kDa) was cloned. PgluE8 efficiently hydrolyzed barley-β-d-glucan followed by CMC-Na, soluble starch, laminarin, and glucan from black yeast optimally at pH 5.5 and 50 °C, and retained 78.6, 41.6, and 34.5 % maximum activity when assayed at 20, 10, and 0 °C, respectively. Enzyme activity remained above 176.6 % after treatment with 10.0 mM β-mercaptoethanol, and was 83.0, 78, and 56 % after pre-incubation in 30 % (w/v) NaCl, 16.67 mg/mL trypsin, and 160.0 mg/mL protease K, respectively. Cys23 and Cys364 residues were critical for PgluE8 activity. *pglue8*, identified from *B. frontalis* feces for the first time in this study, is a potential alternative for applications including food processing, washing, and animal feed preparation.

## Background

Cellulose is considered to be the most abundant renewable resource (Xiang et al. 2014). β-Glucans, which are homopolymers of d-glucose linked by β-glycosidic bonds, commonly exist as cellulose in plants, the bran of cereal grains, and the cell walls of yeast, fungi, and bacteria (Na et al. 2015). Cellulose is solubilized by cellulases, which can be classified into the following three categories per catalytic mechanism: endoglucanase (E.C.3.2.1.4), exoglucanase (E.C.3.2.1.91) and β-glucosidase (E.C.3.2.1.21) (Javed et al. 2009). Endoglucanases are usually involved in the initial stages of cellulose breakdown and act synergistically with exoglucanase and β-glucosidase during the solubilization of cellulose, which is an essential step in the bioprocessing of plant lignocellulosic materials into sugars (Yennamalli et al. 2011). It has been reported that lignocellulosic biomass can be converted to energy, feed, chemical raw materials via microbe-based technology (Zhou et al. 2012; Kumar et al. 2008). Endoglucanases, which have limited ranges of temperature and pH (Li et al. 2009), widely exist in nature and are extracted from bacteria, animals, and fungi, particularly from *Trichoderma*, *Aspergillus,* and *Penicillium* (Xiang et al. 2014). However, several bacterial endoglucanase genes have been isolated from many *Bacillus* species from various environments, such as *B. subtilis* (Qiao et al. 2009; Jung et al. 2010; Furtado et al. 2011), *B. licheniformis* (Teng et al. 2006), *B. halodurans* (Akita et al. 2005), *B. circulans* (Bueno et al. 1990), *B. altitudinis* (Mao et al. 2013), a *Bacillus* sp. (Kim et al. 2013), and *Paenibacillus* sp. (Na et al. 2015; Cheng et al. 2014). Many of them were found to be potential new tools for industrial applications.

Previous reports have indicated that endoglucanase may have potential applications in the food processing, textile, paper, animal feed, and renewable energy industries (MacDiarmid and Venancio 2006). However, these technologies have hardly been practically realized because of the high running cost and low enzyme yields (Li et al. 2009; Limauro et al. 2001; Ando et al. 2002; Huang and Monk 2004; Ng et al. 2009). Therefore, screening of enzyme activity of high endoglucanase has great value.

*Bos frontalis* is one of the most precious and endangered species of wild animals in China. They are widely distributed in Tibet and along the Nujiang river of Yunnan province in China, and they live in primitive forests at altitudes of 2000 m. Bamboo and weeds are the main constituents of their diet and thus it is likely that they harbor unique gastrointestinal microorganisms that possibly produces a novel cellulases. Thus far, the genes encoding this cellulases in *Bos frontalis* have not been reported.

Therefore, in this study, a strain of cellulase-producing bacterium was isolated from *Bos frontalis* feces and the endoglucanase gene was cloned. The gene was expressed in *Escherichia coli*, and the purified recombinant enzyme was characterized and showed tolerance to NaCl, protease and cold.

## Methods

### Vectors and reagents

*Paenibacillus* sp. YD236 was isolated from *Bos frontalis* feces from the Nujiang river of the Yunnan province, China. The taxon for YD236 was identified by 16S rDNA sequencing using the following primers: 27F and 1492R (Table 1).

**Table 1:** Primers used in this study

Primer name	Primer sequence (5′ → 3′)^a^	Tm (°C)
27F	AGAGTTTGATCCTGGCTCAG	52
1492R	GGTTACCTTGTTACGACTT	
*Pglu8*F	GCCTGTACCTGGCCTGCCTG	65
*Pglu8*R	GTGTGAATTTGCGCATTCCTGG	
*E55A*F	TCGCAAAATCACCACCTCCGCAGGGCAAAGTTACG	55
*E55A*R	TGCGGAGGTGGTGATTTTGCGAGCGTCGCTGGGGTCGATG	
*D116A*F	GGACAGCAATTCGGCCTCCGCTGGTGATGTCTGGATG	55
*D116A*R	AGCGGAGGCCGAATTGCTGTCCAGCACTTCCCAC	
*C23G*F	ATATGGAATTGGCCCTTGCCGGTACCTGGCCTGCCTG	55
*C23G*R	ACCGGCAAGGGCCAATTCCATATGTATATCTCCTTC	
*C364G*F	TACCTGACTGGGGCCAGGAAGGCGCAAATTCACAC	55
*C364G*R	GCCTTCCTGGCCCCAGTCAGGTAATAACTCACCTTTTG	

*Tm* annealing temperature

^a^IUPAC/IUB symbols are used

*E. coliTrans*1-T1 (TransGen, Beijing, China) was used for gene cloning, while the *pEASY*-E2 Expression Kit and *E. coli* BL21 (DE3) (TransGen) were used for gene expression. The Fast Mutagenesis System (TransGen, Beijing, China) was used for site-directed mutagenesis. The plasmid isolation kit was purchased from OMEGA (USA). Nickel-NTA agarose (Qiagen, Valencia, CA) was used for the His-tagged protein purification. DNA polymerases (*rTaq* and *LA Taq*) and dNTPs were purchased from TaKaRa (Otsu, Japan). Barley-β-d-glucan was purchased from Sigma (St. Louis, MO), laminarin, glucan from black yeast, cellobiose, cellotriose, cellotetraose and cellopentaose were obtained from J&K (Beijing, China), and TLC Silica gel 60 plates were from Merck KGaA (Germany). All other reagents were of analytical grade and were commercially available.

### Microorganism isolation and identification

*Bos frontalis* fecal samples were collected from the Nujiang river in the Yunnan province, China. Fecal samples (10 g) were suspended in 0.7 % (w/v) NaCl and spread onto screening agar plates containing 0.2 % (w/v) carboxymethyl cellulose sodium salt (CMC-Na), 2 % (w/v) soluble starch, 0.02 % (w/v) Congo red, 0.1 % (w/v) KNO_3_, 0.05 % (w/v) NaCl, 0.05 % (w/v) K_2_HPO_4_, 0.05 % (w/v) MgSO_4_, 0.001 % (w/v) FeSO_4_·7H_2_O, 2 % (w/v) Agar, at pH ranging between 7.2 and 7.4.

Pure culture of the YD236 strain was obtained through repeated streaking on the screening agar plates at 37 °C.

### Genome sequencing

Here, the YD236 genome was sequenced as follows:

#### Library preparation

YD236 genomic DNA was extracted using the OMEGA genomic DNA Isolation Kit, and assessed for quality using the NanoDrop-2000 (Thermo Scientific, Waltham, MA), quantified using the Qubit DNA Quantification Kit (Invitrogen, Carlsbad, CA), randomly fragmented using the Bioruptor sonicator (Diagenode, Liège, Belgium), and purified using Zymo Genomic DNA Clean and Concentration Kit (Orange, CA). The DNA library was then prepared using the NEBNext Ultra DNA Library Prep Kit from Illumina (San Diego, CA, USA), per the manufacturer’s instruction. Post adapter ligation, a library fragment size of appropriately 500 bp was chosen and PCR-enriched (for 10 PCR cycles). The library quality and quantity were confirmed using Bioanalyzer 2100 (Agilent, Santa Clara, CA, USA).

#### Sequencing

The MiSeq Reagent Kit V3 (Illumina) provided reagents for cluster amplification and sequencing on the Miseq system.

#### Data analysis

Real-time image analysis and base calling were performed using the compatible sequencing software RTA (Illumina). FASTAQ files were generated using CASAVA (Illumina) and loaded onto Velvet 1.2.07 for sequence assembly performed on a NF supercomputing server (Inspur, Shandong, China) (Zerbino and Birney 2008).

### Sequence and structure analyses of endoglucanase

Genes and ORFs were predicted, and the glycoside hydrolase family of proteins was classified using dbCAN (http://csbl.bmb.uga.edu/dbCAN/annotate.php). Signal peptides and domains in PgluE8 were predicted using SignalP (http://www.cbs.dtu.dk/services/SignalP/) and the Pfam online tool (http://pfam.xfam.org/), respectively. Identity values of DNA and protein sequences were calculated using BLASTN and BLASTP, respectively (http://blast.ncbi.nlm.nih.gov/Blast/). Multiple sequence alignment was performed using ESPritpt 3.0 (http://espript.ibcp.fr/ESPript/cgi-bin/ESPript.cgi). Model building was performed using the SWISS-MODEL and Swiss-Pdb Viewer programs (http://www.expasy.ch/spdbv). Phylogenetic tree construction were performed using MEGA6.0 (Tamura et al. 2013). Other sequential analyses were performed using the Vector NTI 10.3 software (InforMax, Gaithersburg, MD).

### Expression of *pglue8* in *E. coli*

The coding sequence (CDS) of *pglue8* was amplified by PCR using the *LA Taq* DNA polymerase and the primers *Pglu8*F and *Pglu8*R (Table 1). The resulting PCR product was directly cloned into the *pEASY*-E2 vector by T-A ligation. The recombinant plasmid (*pEASY*-E2-*pglue8*) was transformed into *E. coli* BL21 (DE3) competent cells, and the positive transformants were subsequently identified by PCR analysis and confirmed by DNA sequencing. The transformant harboring *pEASY*-E2-*pglue8* was picked up from a single colony and grown overnight at 37 °C in Luria–Bertani medium containing 0.1 mg/mL ampicillin. The culture was then inoculated at a 1:100 dilution into a culture-complex auto-inducing media (CAI) containing ampicillin and was cultured further at 25 °C for 24 h (Studier 2005).

### Purification and identification of recombinant PgluE8

Cells were harvested by centrifugation at 5000×*g* at room temperature for 5 min and resuspended in McIlvaine buffer (pH 7.0). Cells were disrupted by sonication (7 s, 150 W) on ice several times and centrifuged at 13,000×*g* for 10 min at 4 °C. The supernatant was applied to a Ni^2+^-NTA agarose gel column for purification using a linear imidazole gradient of 20–500 mM in buffer A [20 mM Tris–HCl, 0.5 M NaCl, 10 % (v/v) glycerol, pH 7.2]. The purified protein was detected by 12 % sodium dodecyl sulfate–polyacrylamide gel electrophoresis (SDS-PAGE), and the in-gel band was excised and verified using matrix-assisted laser desorption/ionization time-of-flight mass spectrometry (MALDI-TOF MS) performed by Tianjin Biochip (Tianjin, China).

### Enzyme assay

Endoglucanase activity was determined by measuring the release of reducing sugars from the substrate. A standard reaction comprised 900 μL of 0.7 % (w/v) substrate in Mcllvaine buffer (pH 5.5) and 100 μL of an appropriately diluted enzyme. After incubation at 37 °C for 10 min, the reaction was stopped using 1.5 mL of 3,5-dinitrosalicylic acid (DNS) reagent and the mixture was boiled at 100 °C for 5 min to produce a reddish-brown product quantifiable at 540 nm. One unit (U) of endoglucanase activity was defined as the amount of enzyme that catalyzed formation of 1.0 μmol of reducing sugars equivalent to glucose per minute. Enzyme activity was assayed by following this standard procedure unless otherwise noted.

### Biochemical characterization

To characterize purified Pglu8 activity, various substrates including 0.7 % (w/v) CMC-Na, soluble starch, barley-β-d-glucan, laminarin, and glucan from black yeast (determined at pH 5.5 and 50 °C) were added to each reaction solution. Any further biochemical characterization were performed using barley-β-d-glucan as the substrate.

Optimal pH for endoglucanase activity of purified PgluE8 was determined at 37 °C in different buffers with pH values ranging from 2.0 to 12.0. pH stability of the enzyme was determined by incubating the enzyme solution in various buffers at 37 °C for 1 h. The buffers used were McIlvaine buffer (pH 2.0–8.0), 0.1 M Tris–HCl (pH 8.0–9.0) and 0.1 M glycine-NaOH (pH 9.0–12.0). The residual enzyme activity was measured under the standard assay conditions.

The optimal temperature for the activity of purified PgluE8 was determined over a range of 0–80 °C in McIlvaine buffer (pH 5.5). Thermostability of the enzyme was determined under standard assay conditions following pre-incubation of the enzyme for 1 h at 37, 50, or 60 °C, with the untreated enzyme defined as having 100 % activity.

Effects of different metal ions and organic reagents on the endoglucanase activity were determined in McIlvaine buffer (pH 5.5) at 50 °C. We added 1.0 or 10.0 mM (final concentration) CaCl_2_, NiSO_4_, CoCl_2_, MgSO_4_, KCl, ZnSO_4_, FeCl_3_, Pb(CH_3_COO)_2_, MnSO_4_, FeSO_4_, HgCl_2_, EDTA, β-mercaptoethanol, SDS, and 3.0–30.0 % (w/v) NaCl to the reaction solution. A reaction without metal ions or organic reagents was used as a control.

To examine its resistance to different proteases, 0.234 mg purified PgluE8 was incubated at 37 °C for 1 h with 9.09–66.67 mg/mL trypsin (pH 7.3) or 16.67–160 mg/mL proteinase K (pH 7.3). Effects of 3.0–30.0 % (w/v) NaCl (pH 7.5) on the endoglucanase activity were determined in McIlvaine buffer (pH 5.5) at 37 °C. To determine its resistance to salt stress, PgluE8 was incubated with 3.0–30.0 % (w/v) NaCl (pH 7.5) at 37 °C for 1 h. The residual enzyme activity was measured in the buffer and at temperatures corresponding to treated condition.

*K*_*m*_, *V*_*max*_, and *k*_*cat*_ values for purified PgluE8 were determined using 0.05–1.5 % (w/v) barley-β-d-glucan as the substrate in McIlvaine buffer (pH 5.5) at 50 °C. Data were plotted according to the Lineweaver–Burke method (Zhou et al. 2012).

### Hydrolysis products

Hydrolysis of barley-β-d-glucan (0.7 % w/v; pH 5.5) was performed with a reaction system of 1 U/mL purified PgluE8 at 50 °C for 1 and 3 h. Hydrolysis products were analyzed by thin layer chromatography (TLC) as previously described (Zhou et al. 2014). Glucose, cellobiose, cellotriose, cellotetraose and cellopentaose mixture were used as standards. Barley-β-d-glucan with the inactivated PgluE8 (100 °C for 5 min) was used as a control.

### Site-directed mutagenesis

Site-directed mutagenesis was performed using the Fast Mutagenesis System (TransGen) using the following primers (modified codons underlined): *E55A*F and *E55A*R for the E55A mutant; *D116A*F and *D116A*R for the D116A mutant; *C23G*F and *C23G*R for the C23G mutant; *C364G*F and *C364G*R for the C364G mutant (Table 1). DNA manipulations were performed according to the manufacturer’s instructions. All mutation sites were confirmed by DNA sequencing. For expression, plasmids were transformed into *E. coli* BL21 (DE3) cells. Activity of the enzyme variants was determined at pH 5.5 and 50 °C. The purified PgluE8 was used as a control.

### Nucleotide sequence accession numbers

Nucleotide sequences for the *Paenibacillus* sp. YD236 16S rDNA and endoglucanase gene (*pglue8*) were deposited in GenBank under accession numbers KR071621 and KR150023, respectively.

## Results

### Strain identification

The comparison of the 16S rDNA sequence from YD236 (1458 bp,KR071621) with those in GenBank yielded nucleotide identities of 99 % with *Paenibacillus amylolyticus* strain KT5501 (AB115960), 98 % with *Paenibacillus amylolyticus* strain MLL-8 (JQ956529), and 98 % with *Paenibacillus* sp. 7B-841 (KF441702). Thus, the strain YD236 was classified into the genus *Paenibacillus*.

### Genome sequencing and sequence analyses

**Fig. 1:**
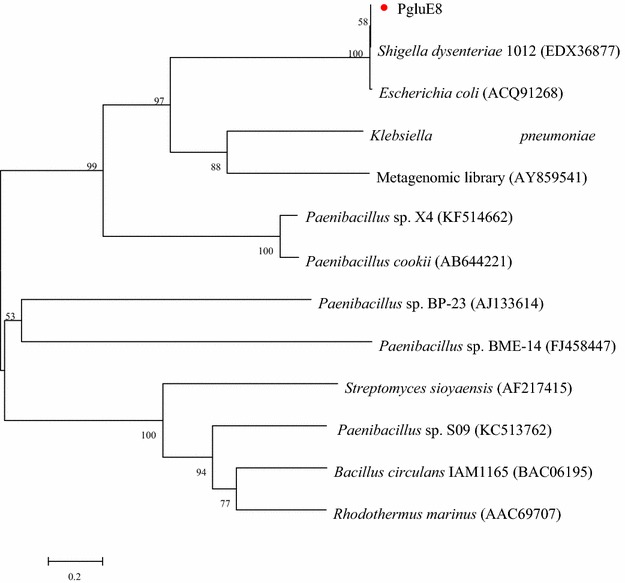
Phylogenetic tree constructed using the neighbor-joining method based on the amino acid sequences of endoglucanase from different strains

Genome sequencing generated 470 Mbp of sequence data, which was 7.6 Mbp after sequence assembly. Local BLAST analysis revealed that the gene *pglue8* was homologous to the glycosyl hydrolase family 8 (GH8) enzyme. Phylogenetic and BLASTp analyses PgluE8 (Fig. 1) with another twelve endoglucanase from different strains. BLASTp analysis showed that PgluE8 shared the highest identities with the cellulase from *Shigella dysenteriae* 1012 (EDX36877; 100 %) and with the cellulase from *E. coli* (ACQ91268; 99 %) (Li et al. 2009). Following that, PgluE8 showed 14.89, 3.68 and 5–25 % amino acid sequence identity with GH8 β-1,3-1,4-glucanase from *Paenibacillus* sp. X4 (Na et al. 2015), GH16 β-1,3-1,4-glucanase from *Paenibacillus* sp. S09 (Cheng et al. 2014), and with other endoglucanase, respectively.

The full-length *pglue8* (1107 bp) starts with the putative codon ATG and ends with TAA, encoding a 368 residues polypeptide with a calculated mass of 40.4 kDa. The signal peptide was predicted from M1 to A21.

**Fig. 2:**
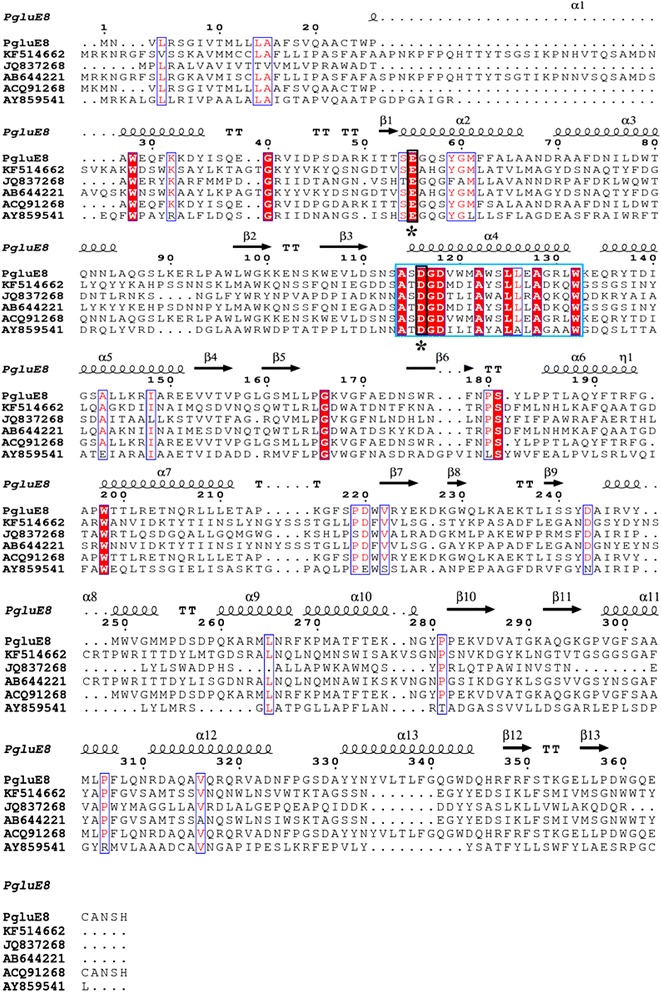
Partial amino acid sequence alignment of PgluE8 with glycosyl hydrolase family 8 endoglucanases. Sequence names, except PgluE8, are shown with accession numbers as follows: BGlc8H from *Paenibacillus* sp. X4 (KF514662), Cel8A from *Klebsiella pneumoniae* (JQ837268), Pgl8A from *Paenibacillus cookie* (AB644221), and Cel124 from a metagenomic library (AY859541). Conserved residues indicated by *black line frames*. *Asterisks* indicate putative catalytic residues. *Symbols* above the sequences indicate the secondary structure

By comparison with the GH8 endoglucanase BGlc8H (KF514662) (Na et al. 2015), Cel8A (JQ837268) (Ng et al. 2013), Pgl8A (AB644221) (Shinoda et al. 2012) and Cel124 (AY859541) (Xiang et al. 2014), the multiple sequence alignment of PgluE8 revealed a sequence from A114 to W132 amino acid residues (Fig. 2). The highly conserved residues necessary for the catalytic activities of GH8 enzymes have been reported to be one glutamate and one aspartate (Shinoda et al. 2012), and the active sites of the β-1,3-1,4-glucanase of *Paenibacillus* sp. X4 are Glu95 and Asp156 (KF514662) (Na et al. 2015). In this study, the site-directed mutants (Table 3), E55A and D116A, of PgluE8 showed nearly complete loss of enzyme activity, indicating that Glu55 and Asp116 are the catalytically active residues of PgluE8.

### Expression, purification, and identification of PgluE8

**Fig. 3:**
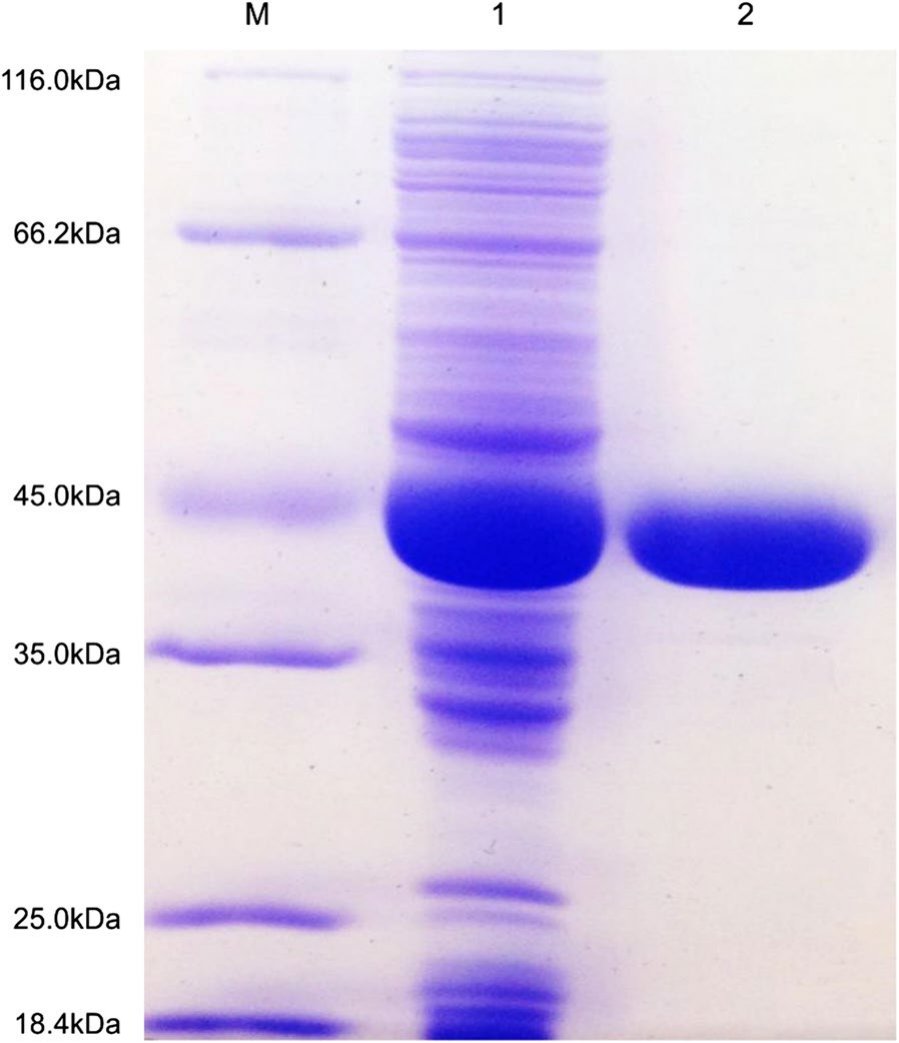
SDS-PAGE analyses of PgluE8. *Lanes*: *M* low-molecular weight markers; *1* crude PgluE8; and *2* PgluE8 purified by Ni^2+^-NTA affinity chromatography

**Fig. 4:**
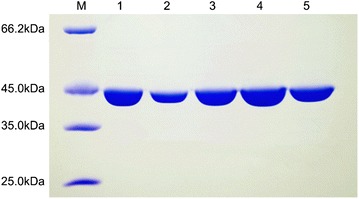
SDS-PAGE analyses of enzyme of site-directed mutagenesis. *Lanes*: *M* low-molecular weight markers, *1* purified Glu34, *2* purified Asp 95, *3* purified Cys2, *4* purified Cys343, *5* purified Cys2 and Cys343 double mutants

PgluE8 were expressed in *E. coli* BL21 (DE3) (Fig. 3). PgluE8 and enzyme of site-directed mutagenesis (Fig. 4) were purified to electrophoretic homogeneity by Ni^2+^-NTA metal chelating affinity chromatography. The purified PgluE8s migrated as single bands during SDS-PAGE with molecular masses of approximately 35 and 45 kDa, respectively, which are close to the calculated values (PgluE8: 40.4 kDa). After tryptic digest, PgluE8s were individually analyzed using MALDI-TOF MS. Results revealed that the MALDI-TOF MS spectra matched the molecular masses of the known internal peptides of PgluE8 (full data analysis was not shown), confirming that the purified enzymes were PgluE8.

### Substrate specificity

At pH 5.5 and 50 °C, the activities of the purified PgluE8s for 0.7 % (w/v) CMC-Na, soluble starch, barley-β-d-glucan, laminarin, and glucan from black yeast were 2.3 ± 0.3, 0.9 ± 0.3, 11.0 ± 0.3, 0.8 ± 0.1 and 0.7 ± 0.1 U/mg, respectively.

### Enzyme characterization

**Fig. 5:**
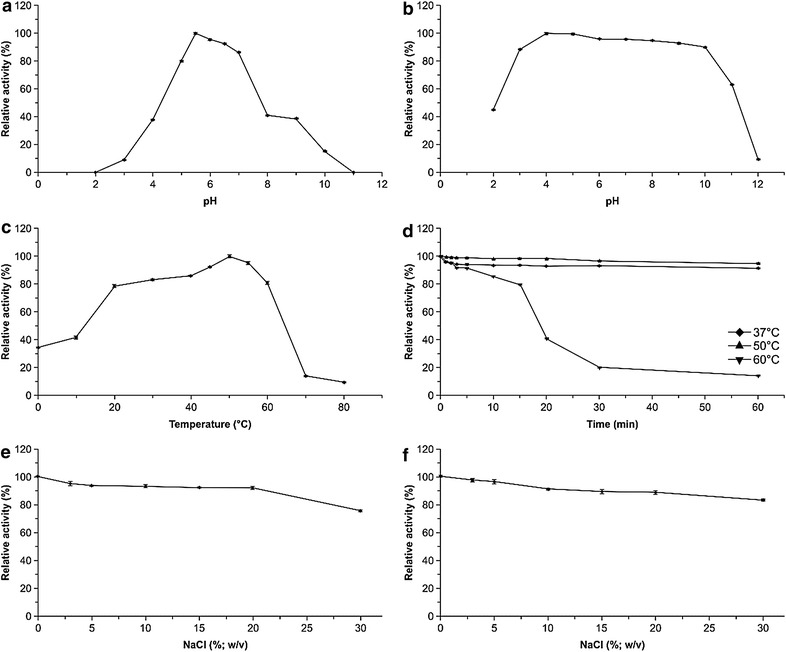
Characterization of purified PgluE8. **a** Effect of pH on PgluE8, **b** pH stability of PgluE8, **c** effect of temperature on PgluE8, **d** thermostability of PgluE8, **e** effect of NaCl on purified PgluE8 at pH 5.5 and 37 °C, **f** stability of PgluE8 in NaCl

When assayed at 37 °C the purified PgluE8 showed an apparently optimal endoglucanase activity at pH 5.5 retaining greater than 80 % of its maximum activity between pH 5.0 and 7.0 (Fig. 5a). The pH stability assay showed that at pH ranging from 3.0 to 11.0, the purified PgluE8 exhibited more than 63 % of its initial activity (Fig. 5b).

When assayed at pH 5.5, the purified PgluE8 was optimal at 50 °C, retaining 78.6, 41.6, and 34.5 % of its maximum activity when assayed at 20, 10, and 0 °C, respectively (Fig. 5c). The enzyme was stable at 37 and 50 °C for up to 1 h, exhibiting 91.5 and 94.7 % of its maximum activity, respectively. Half-lives of the enzyme ranged from 15 to 20 min at 60 °C (Fig. 5d).

The activity of the purified PgluE8 was strongly or partially inhibited by 1.0 and 10.0 mM Ag^+^ (retaining 9.4 % activity), Hg^2+^ (retaining 10.8 % activity) and SDS (retaining 29.7 % activity), and partially inhibited by Fe^2+^, Mn^2+^, Fe^3+^ and Pb^2+^. In contrast, β-mercaptoethanol (retaining 176.6 % activity), K^+^ (retaining 128.7 % activity), Na^+^ (retaining 125.4 % activity) and Mg^2+^ (retaining 125.0 % activity) enhanced the activity. The addition of other reagents showed little or no effect on the enzymatic activity (Table 2).

**Table 2:** Effect of metal ions and organic reagents on the activity of purified PgluE8

Reagent	Relative activity (%)^a^	
	10 mM	1 mM
None	100.0 ± 0.9	100.0 ± 0.1
β-Mercaptoethanol	176.6 ± 0.9	115.0 ± 0.5
KCl	128.7 ± 0.5	98.7 ± 0.3
NaCl	125.4 ± 0.6	98.2 ± 0.2
MgSO_4_	125.0 ± 0.3	99.7 ± 0.5
CoCl_2_	108.8 ± 1.3	102.8 ± 0.2
CuSO_4_	104.5 ± 1.1	95.2 ± 0.2
CaCl_2_	100.8 ± 1.1	97.3 ± 1.4
EDTA	98.5 ± 0.4	87.4 ± 0.9
ZnSO_4_	84.4 ± 0.7	73.5 ± 0.7
NiSO_4_	84.1 ± 1.1	88.9 ± 0.1
FeSO_4_	77.8 ± 0.5	87.3 ± 0.3
MnSO_4_	74.2 ± 0.8	95.6 ± 0.1
FeCl_3_	72.8 ± 0.6	95.0 ± 0.3
PbAc	59.3 ± 0.7	78.6 ± 0.4
SDS	29.7 ± 0.9	11.2 ± 0.6
HgCl_2_	10.8 ± 0.3	12.6 ± 0.3
AgNO_3_	9.4 ± 0.3	10.2 ± 1.0

^a^Values represent the mean ± SD (n = 3) relative to the untreated control samples

The activities of the enzyme variants Cys23 and Cys364 were 148.1 ± 0.3 and 140.5 ± 0.2 %, respectively, and for the Cys23 and Cys364 double mutants the activity was 155.2 ± 0.4 % (Table 3).

**Table 3:** Enzyme activity of wild and mutant type recombinant PgluE8 E55A, PgluE8 D116A, PgluE8 C23G, PgluE8 C364G and PgluE8 C23G-C364G

Enzyme	Activity (U/mg)	Relative activity (%)^a^
Wild type PgluE8	5.86 ± 0.23	100.00
PgluE8 E55A	0.52 ± 0.12	8.87
PgluE8 D116A	0.71 ± 0.09	12.12
PgluE8 C23G	8.60 ± 0.30	148.08
PgluE8 C364	8.24 ± 0.13	140.46
PgluE8 C23G-C364G	9.10 ± 0.42	155.28

^a^Values represent the mean ± SD (n = 3) relative to the untreated control samples

The purified PgluE8 exhibited good NaCl activity, retaining 94.8–75.2 % activity at concentrations between 3.0 and 30.0 % (w/v) NaCl (Fig. 5e). It also retained 97.3–83.1 % of its initial activity following incubation with 3.0–30.0 % (w/v) NaCl at 37 °C for 1 h (Fig. 5f).

**Fig. 6:**
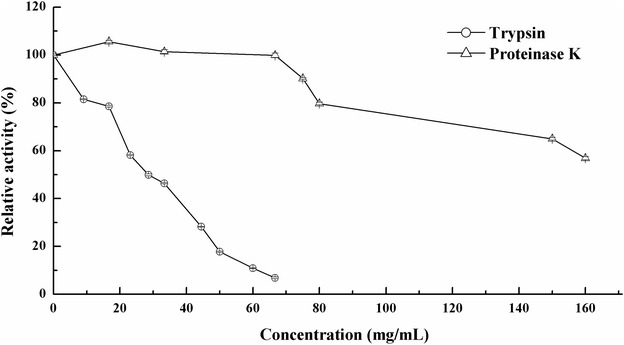
Stability of PgluE8 in various concentrations of trypsin and proteinase K at 37 °C for 1 h at pH 7.3. The residual enzyme activity was determined in McIlvaine buffer (pH 5.5) at 37 °C

The purified PgluE8 could be tolerant to up to 16.67 mg/mL trypsin, 80.0 mg/mL proteinase K and 160.0 mg/mL proteinase K retaining more than 78, 79 and 56 % of its initial activity, respectively. However, almost all PgluE8 activity was lost after incubation with 66.67 mg/mL trypsin at 37 °C for 1 h (Fig. 6).

Based on a Lineweaver–Burk plot, the *K*_*m*_, *V*_*max*_, and *k*_*cat*_ values were 10.5 mg/mL, 45.7 U/mg, and 31.7 s^−1^, respectively, with barley-β-d-glucan as the substrate.

### Hydrolysis products

**Fig. 7:**
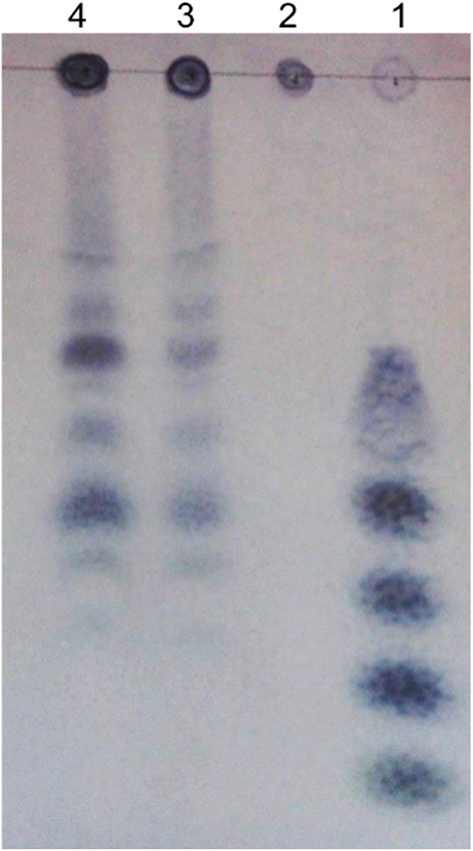
Thin layer chromatography (TLC) of hydrolysis products of 0.7 % (w/v) barley-β-d-glucan. *Lanes*: *1* glucose, cellobiose, cellotriose, cellotetraose, and cellopentaose markers; *2* barley-β-d-glucan with inactivated (at 100 °C for 5 min) purified PgluE8; *3* barley-β-d-glucan hydrolyzed by purified PgluE8 for 1 h in a 1.0 U/mL reaction system at 50 °C and pH 5.5; *4* barley-β-d-glucan hydrolyzed by purified PgluE8 for 3 h in a 1.0 U/mL reaction system at 50 °C and pH 5.5

The hydrolysis products of 0.7 % (w/v) barley-β-d-glucan were analyzed by TLC (Fig. 7). Cellotriose, cellotetraose, and cellopentaose were released from barley-β-d-glucan by the purified PgluE8, while glucose and cellobiose, were not clearly detected. The hydrolysis analysis indicated the endo-acting nature of PgluE8.

## Discussion

Cellulase is one of the most important enzymes used for the production of bioenergy from lignocellulosic biomass. Although the activity of fungal cellulase was greater than that of bacterial cellulase, the use of bacterial cellulase overcomes challenges of thermostability and allows for activity over a wide pH range and for broader substrate utilization (Maki et al. 2009). To the best of our knowledge, this study is the first to report a endoglucanase gene from *Bos frontalis* feces, PgluE8, which showed good enzyme characteristics.

Genome sequencing has now become both rapid as well as cost-efficient. Some enzymes showing important values for basic research and industrial application were obtained based on genome sequencing (Chen et al. 2013; Song et al. 2014; Sakka et al. 2012). Sequence analysis showed that PgluE8 was the most similar to GH8 endoglucanase. The optimal temperature of PgluE8 was found to be similar to that reported for cellulolytic enzymes and it was stable in a from 30 to 55 °C (Xiang et al. 2014). Cold-active enzymes are highly active at low temperatures (Siddiqui and Cavicchioli 2006). Bhat reported that CEL8M showed 28 % enzyme activity at 10 °C (Bhat et al. 2013). Compared with CEL8M, PgluE8 exhibited much higher endocellulase activity at both 10 °C (41.6 %). The PgluE8 even showed 34.5 % of its maximum activity at 0 °C. The mechanism that correlate with enzymatic adaptations to low temperatures have attracted considerable research attention (Siddiqui and Cavicchioli 2006; Collins et al. 2002). For instance, cold-active enzymes generally possess less numbers of P amino acids (Siddiqui and Cavicchioli 2006), which generally can increase the stability of the protein. Besides, increasing frequency of the charged (H, D, E) and hydrophobic amino acids likely lead to enhance the thermo adaptation of protein. Therefore, compared with the hyperthermophilic and thermophilic endoglucanase (Table 4), PgluE8 has less numbers of P, H and hydrophobic amino acids. These changes might result in a reduced stability and an enhanced flexibility of the molecular structure, thereby enabling the cold-active endoglucanase activity of PgluE8. Meanwhile, it is worth mentioning that PgluE8 preformed maximal activity under acidic conditions. Its optimal pH value is 5.5, compared to the endo-1, 4-β-glucanase from *Ruminococcus albus* at pH 6.8 (Deguchi et al. 1991), the endoglucanase Ss from *C. thermocellum* at pH 6.6 (Fauth et al. 1991), the endoglucanase from *Volvariella volvacea* at pH 7.5 (Ding et al. 2002), and the endoglucanase C from *Clostridium cellulolyticum* at pH 6.0 (Fierobe et al. 1993). The pH stability assay showed that at pH ranging from 3.0 to 11.0, the purified PgluE8 exhibited more than 63 % of its initial activity. The stable pH rang for PgluE8 was wider than those for most endoglucanase from other strains, such as *Bacillus* sp. KSM-635 (pH 6–11) (Yoshimatsu et al. 1990) and *Bacillus* sp. KSM-S237 (pH 5–11) (Hakamada et al. 1997). To the best of our knowledge, the mechanism of stable pH rang for endoglucanase analysis have not been reported. This property suggests that PgluE8 could have great potential applications in the field of feed additives. The results show that PgluE8 has the lowest temperature and pH optima compared to previously identified enzymes. Hence, it can be used for low temperature washing, biopolishing of cotton-based fabric to remove fuzz at low temperatures, and finishing denims by stone washing using cellulase at low pH and low temperature in textile industry (Bhat et al. 2013).

**Table 4:** Partial sequence analysis and enzyme characterization of thermophilic, cold-active and NaCl-tolerant endoglucanase from different strains

Endoglucanase	Cel5H	Rucel5B	celVA	PgluE8
Optimum temperature	50–85 °C	60	80	55
Relative activity (%) at 0 °C	NR	NR	NR	34.5
Relative activity (%) at 10 °C	NR	NR	NR	41.6
Relative activity (%) at 20 °C	NR	NR	NR	78.6
Optimum pH	5.0	6.5	3.6–4.5	5.5
pH stability	4.6–5.6 (80 %)	NR	NR	3.0–10.0 (>88 %)
NaCl-tolerant	4 M (70 %)	NR	NR	30 % (ca. 5 M) (83 %)
P (Pro)^a^	5.45	5.65	6.41	5.43
H (His)^a^	2.88	1.79	2.03	0.54
Hydrophobic AA^a^	39.43	42.26	44.23	39.13
Total ASA	13,645.64	12,450.21	21,411.93	13,701.91
Exposed nonpolar ASA	8224	7616.52	13,451.81	7541.92
Total volume (packing)	42,258	41,660.11	61,825.52	46,735.51
Nonpolar relative ASA	0.61	0.62	0.63	0.55
Accession no.	ACI19154	GQ849224	LN626709	KR150023
Organism	*Dictyoglomus thermophilum*	Yak rumen metagenome	*Alicyclobacillus vulcanalis*	This study
Reference	Shi et al. (2013)	Bao et al. (2011)	Boyce and Walsh (2015)	This study

*Hydrophobic AA* hydrophobic amino acids A, F, G, I, W, P and V, *NR* not reported

Nonpolar relative AS = exposed nonpolar ASA/total ASA

^a^% by frequency

PgluE8 was strongly or partially inhibited by 1.0 and 10.0 mM Ag^+^, Hg^2+^ and SDS, and partially inhibited by Fe^2+^, Mn^2+^, Fe^3+^ and Pb^2+^. However, β-mercaptoethanol (176.6 %), K^+^ (128.7 %), Na^+^ (125.4 %) and Mg^2+^ (125.0 %) enhanced the activity. Thus, the enzyme possibly belongs to the class of SH enzymes and β-mercaptoethanol probably keeps the enzyme in a reduced state thereby increasing the activity. Similar observations have been reported for the endoglucanases of *Fusarium oxysporum* (Christakopoulos et al. 1995) and *Penicillium purpurogenum* (Lee et al. 2010). Notably, the activities of enzyme variants were strongly enhanced. Site-directed mutagenesis further confirmed that Cys23 and Cys364 play important roles in the activity. This result advances the understanding of the role of the disulfide bond in this class of enzyme, and the mechanism by which β-mercaptoethanol increases catalytic activity.

We have previously investigated salt-tolerant enzymes in great detail (Zhou et al. 2012, 2014, 2015), which showed 127.9–88.4 % activity at concentrations between 3.5 and 15.0 % (w/v) NaCl. As revealed in this study, PgluE8 is considerably NaCl-tolerant, retained more than 83 % activity when 30 % (w/v) (ca. 5 M) NaCl was added to reaction system. Compared with a halotolerant endoglucanase Cel5H from *Dictyoglomus thermophilum* (Table 4) (Shi et al. 2013), PgluE8 showed superior tolerance to NaCl. Nonpolar relative ASA PgluE8 is lower than that of Cel5H, Rucel5B and celVA (Table 4). Therefore, high-NaCl solution likely increase interaction between molecular surface and neutral salt ions, further, protect hydration membrane of the protein from hydration by NaCl, ultimately, protein show salt resistance. Salt-tolerant enzyme has potential uses in processing sea foods and other foods with saline contents of 3.5–15.9 % (w/w) NaCl, such as marine algae, pickles, and sauces (Zhou et al. 2012). In such high-salt processed foods, use of PgluE8 may prevent microbial pollution and save energy.

Few protease-tolerant endoglucanase had been reported. Shunichi Akiba et al. (Akiba et al. 1995) reported that endo-,β-1,4-glucanase activity was not affected when incubated with 250 μg/mL proteinase K. However, in this study, PgluE8 was resistant to digestion by up to 16.67 mg/mL trypsin and 80.0 mg/mL proteinase K, showed superior tolerance to proteinase. Proteases are often supplemented in food and feed (Kuddus and Ramteke 2012). Therefore, the protease resistance and low-to moderate-temperature (body temperature of domestic animals and fish) activity suggested PgluE8 may be a new candidate for feed supplements.

In conclusion, this study is the first to report a cold-active and salt tolerant protease endoglucanase from *Paenibacillus* sp. YD236 isolated from the feces of *Bos frontalis*. PgluE8 showed excellent enzyme characteristics and might be an alternative for potential applications in food processing, washing, animal feed preparation. However, PgluE8 shows low stability at high temperatures; therefore, it is important to improve its catalysis at high temperatures.
